# Integral Imaging Display System Based on Human Visual Distance Perception Model

**DOI:** 10.3390/s23219011

**Published:** 2023-11-06

**Authors:** Lijin Deng, Zhihong Li, Yuejianan Gu, Qi Wang

**Affiliations:** 1 School of Artificial Intelligence, Changchun University of Science and Technology, No. 7089, Weixing Road, Changchun 130022, China; denglijin@cust.edu.cn (L.D.); lizhihonggid@163.com (Z.L.); 2 Zhongshan Institute, Changchun University of Science and Technology, No. 16, Huizhan East Road, Zhongsha 528437, China; 3 School of Electronics and Information Engineering, Changchun University of Sciences and Technology, No. 7089, Weixing Road, Changchun 130022, China; wq965146656@163.com

**Keywords:** integral imaging, human visual distance perception, spatial resolution, depth of field, face-eye tracking

## Abstract

In an integral imaging (II) display system, the self-adjustment ability of the human eye can result in blurry observations when viewing 3D targets outside the focal plane within a specific range. This can impact the overall imaging quality of the II system. This research examines the visual characteristics of the human eye and analyzes the path of light from a point source to the eye in the process of capturing and reconstructing the light field. Then, an overall depth of field (DOF) model of II is derived based on the human visual system (HVS). On this basis, an II system based on the human visual distance (HVD) perception model is proposed, and an interactive II display system is constructed. The experimental results confirm the effectiveness of the proposed method. The display system improves the viewing distance range, enhances spatial resolution and provides better stereoscopic display effects. When comparing our method with three other methods, it is clear that our approach produces better results in optical experiments and objective evaluations: the cumulative probability of blur detection (CPBD) value is 38.73%, the structural similarity index (SSIM) value is 86.56%, and the peak signal-to-noise ratio (PSNR) value is 31.12. These values align with subjective evaluations based on the characteristics of the human visual system.

## 1. Introduction

Integral imaging (II) technology refers to a display technology that captures and presents 3D scene information from various viewpoints. In 1908, G. Lippmann introduced a pioneering imaging technology called integral photography. This technology utilizes the reversibility of light to create 3D images that can be viewed without the need for any special aids [1]. However, the quality of 3D images is hindered by limitations in imaging and display device resolution, which has slowed down the development of II technology. Today, display devices have undergone significant advancements in electronic and optical technologies, resulting in their alignment with human visual perception principles. The mechanisms of human stereoscopic perception are being increasingly utilized, from flat displays to stereoscopic displays, and now to interactive displays. Therefore, research on 3D display technology must consider the characteristics of human stereoscopic vision. Combining the principles of human stereoscopic vision with the design and optimization of parameters for II technology can greatly enhance the overall effect of stereoscopic display.

The three parameters of spatial resolution, viewing angle, and depth of field (DOF) serve as important indicators for the display quality of 3D II. Improving these parameters has always been a research focus in II technology [2,3,4,5,6,7,8,9,10,11,12]. Piao Y et al., proposed a method for extending the DOF in II using multi-focus fusion [13]. Wang QH et al., successfully achieved the optical refocusing of objects with a large DOF in II displays by refocusing at any desired depth [14]. Peng et al., introduced a novel structure of a double pinhole/micro-lens array with two center-depth planes, to enhance the depth-of-field of II. This particular structure can be fabricated using a combination of lithography and inkjet printing techniques [15]. These studies focused on enhancing the DOF, but did not address the issue of resolution improvement. In recent years, there have been studies focused on improving both the DOF and resolution simultaneously. Hui Yun et al., have proposed a new technique for 3D passive image sensing and visualization. This technique utilizes both large and small apertures to capture element images simultaneously, reducing diffraction effects and increasing degrees of freedom. As a result, it improves both the lateral resolution and DOF in II [16]. Shitu Ma et al., proposed the use of time-multiplexed convergent backlight technology to improve the spatial bandwidth of II systems. This technology enhances the resolution, DOF, and viewing angle of these systems simultaneously [17]. While these methods can enhance performance in various aspects, they require high-end hardware for both image capture and display stages. Additionally, much of the existing research focuses on analyzing the light field under ideal conditions, neglecting to consider the visual characteristics of the human eye when receiving light.

**Figure 1:**
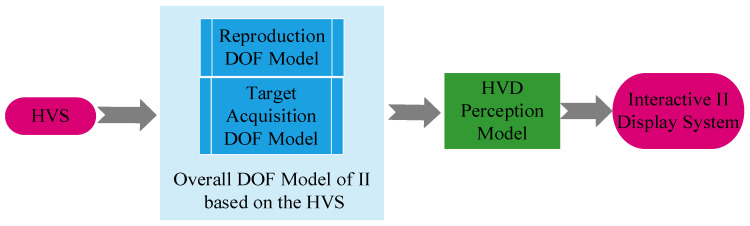
Interactive II display system based on HVD perception model.

The human visual system (HVS) has a certain degree of adaptability to ensure that clear images are focused on the retina. This article analyzes the path of light emitted from a point light source to the human eye in the acquisition and reconstruction of the light field by considering the visual characteristics of the human eye. This research focuses on investigating the reproduction DOF model and the target acquisition DOF model of the II system. Then, an overall DOF model of II is derived based on the HVS. On this basis, an II system based on the human visual distance (HVD) perception model is proposed, and an interactive II display system (Figure 1) is constructed. This display system can enhance the viewing range of the human eye while ensuring spatial resolution and improving stereoscopic display effects.

## 2. Overall DOF Model of II System Based on the Human Visual System

**Figure 2:**
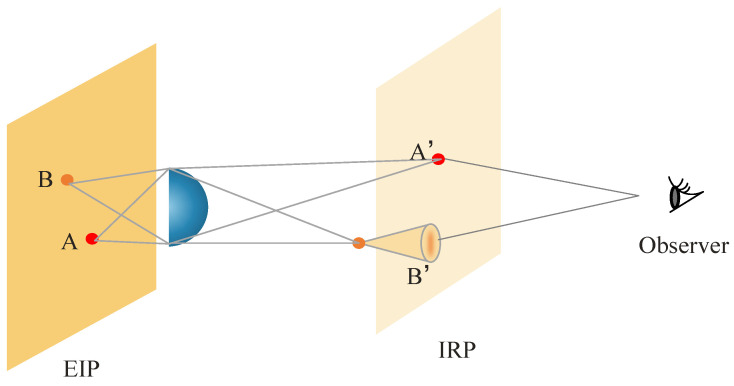
Discrete phenomena occur at off-focus point B.

The element image array (EIA) is composed of element images (EI) that contain 2D images with multiple disparity information, which matches the lens array. The element image panel (EIP) is a display panel used to show EIA images. Each point source on the EIP represents a pixel that contains viewpoint information. The HVS has the ability to adapt and focus clear images onto the retina. Additionally, the sensitivity of spatial resolution varies across different regions of the retina. Point A, located on the focal plane, and point B, located away from the focal plane, are projected as image A and image B onto the EIP. Then, they pass through the lens are formed on the image reference plane (IRP) as A′ and B′. When the human eye focuses on the IRP, only point A is accurately reconstructed as A′. Although the reconstruction plane of point B does not align with the IRP, the HVS has the ability to adjust point B on the IRP. In this scenario, the observer will see a clear spot labeled A′ and a blurry speckle labeled B′, as shown in Figure 2.

In an II display system based on a micro-lens array, the light emitted by pixels on the display screen converges after passing through the micro-lens array. The reconstructed image on the central depth plane (CDP) is the clearest image plane [18]. D represents the distance between the lens array and CDP, while *g* represents the distance between the lens array and EIP. They adhere to the Gaussian formula with focal length (*f*):

(1) $$\frac{1}{g}+\frac{1}{D}=\frac{1}{f}$$

The performance of an II system can be influenced by errors in the size, transmittance, and alignment of the optical components. The discrete phenomena observed at point B, as depicted in Figure 2, will also limit the DOF and spatial resolution during the reconstruction of II. The DOF and spatial resolution of the system are contradictory, and it may be challenging to achieve the desired resolution while maintaining an adequate DOF. The method presented in this research paper endeavors to enhance the DOF for human perception while simultaneously improving spatial resolution.

### 2.1. Reproduction DOF Model

**Figure 3:**
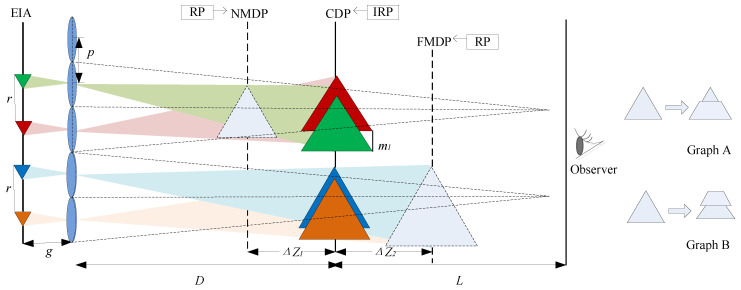
Facet braiding in II display.

In an ideal integrated imaging display system, the reconstructed images generated by each EI through corresponding micro-lenses on the CDP overlap in size and position, enabling observers to view continuous three-dimensional images. However, the reconstruction depth of integrated imaging is limited by factors such as light diffraction and the recognition limit of human visual perception. This limitation leads to facet braiding between unit image planes [19,20], as depicted in Figure 3 for graph A and graph B. Ultimately, this phenomenon results in image ghosting on the visual plane.

Our team has developed a distortion-free reproduction DOF model based on the characteristics of HVS in the II system [21]. The distance from the near margin depth plane (NMDP) to the CDP is:

(2) $$\Delta Z_{1}=\frac{P_{D}D(g+D)}{gp(1+\frac{g^{\prime }}{l^{\prime }})+P_{D}D}$$

The distance from the far margin depth plane (FMDP) to the CDP is:

(3) $$\Delta Z_{2}=\frac{P_{D}D(g+D)}{gp(1+\frac{g^{\prime }}{l^{\prime }})-P_{D}D}$$

*D* represents the distance between the lens array and CDP, while *g* represents the distance between the lens array and EIP and *p* represents the lens’ diameter. The lens’ image distance is *g′*, and the lens’ object distance is *l′*. *P_D_* represents the pixel size of the display device.

Based on an analysis of the facet braiding in the II display caused by human visual characteristics, we have developed a model that provides a more accurate quantification representation of the reconstructed DOF in II systems. This also serves as a foundation for future research on DOF models that take into account HVS.

### 2.2. Target Acquisition DOF Model

**Figure 4:**
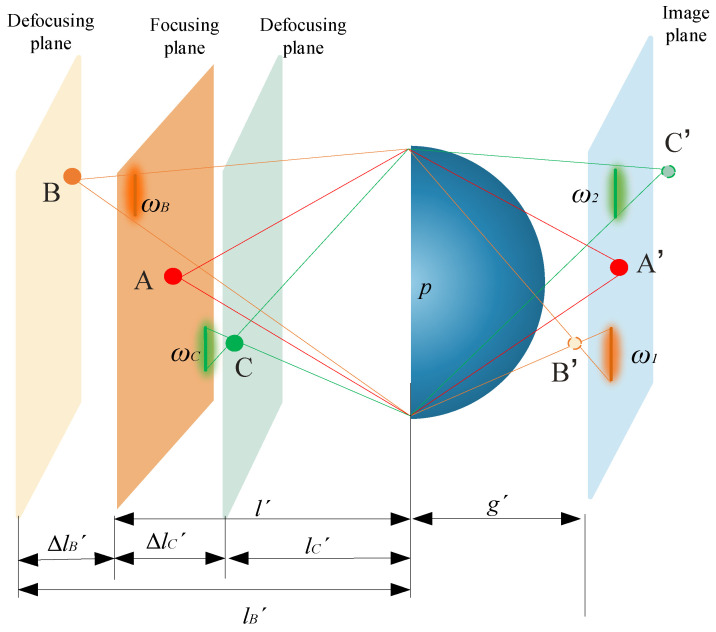
Optical path diagrams of single-lens imaging in the acquisition stage of II.

**Figure 5:**
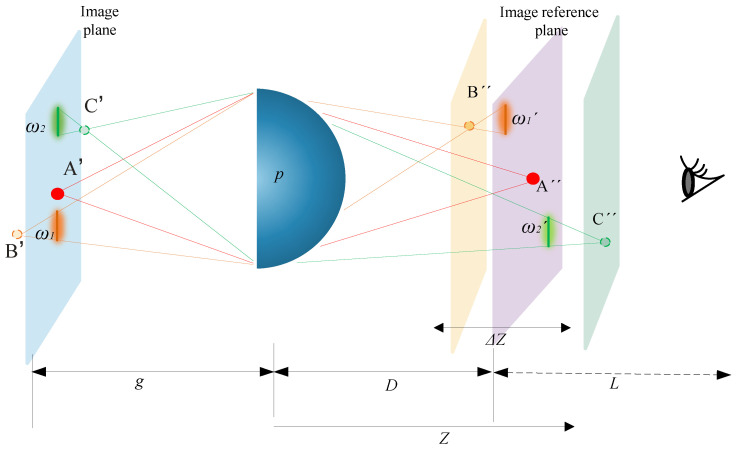
Optical path diagrams of single-lens imaging in the reconstruction stage of II.

Due to the influence of the target acquisition range, only 3D scenes within this range can be clearly recorded and reconstructed into clear 3D images using fixed-parameter acquisition devices [22,23]. The optical path diagrams of single lens imaging in the acquisition stage and reconstruction stage of II are shown in Figure 4 and Figure 5, respectively. The analysis is conducted using reverse ray tracing.

During the acquisition stage of II, the focusing plane in Figure 4 represents the surface where the object distance position of the lens is located, while the image plane represents the surface where the image distance position of the lens is located. Therefore, the object point A located on the focusing plane can be clearly imaged as a light point on the image plane through the lens. However, object points B and C, which are located outside the focal plane (defocusing plane), cannot be clearly imaged as light spots, but instead appear as diffuse spots on the image plane. This is due to the visual characteristics of the human eye, which were introduced in the previous section. During the reconstruction stage of II, all of the image points on the image plane are imaged on the image reference plane through the lens. The corresponding explanation of integral imaging can also be understood as the EIA is imaged on the CDP through the lens. The reconstructed image on the image plane is the clearest.

As depicted in Figure 4, during the acquisition stage of II, the spatial position of point A is located on the focusing plane of the lens, so point A will be clearly imaged as point A’ on the image plane. Since the spatial positions of points B and C are both on the defocusing plane (point B is in front of the focusing plane and point C is behind it), they are far away from the focusing plane of the lens. Eventually, point B and point C will form diffraction spots with diameters *ω_B_* and *ω_C_*, respectively on the focusing plane.

In Figure 5, the image point B’ is located outside of the image plane, the diameter of the diffraction spot formed on the image plane is ω_1_, and the reconstruction stage corresponds to a diffraction spot diameter of *ω_1′_* on the image reference plane. According to the optical path diagrams in Figure 4 and Figure 5, the diffraction spots’ diameter of point B can be obtained as:

(4) $$\left\{\begin{array}{l} \omega_{B}=\frac{l_{B}^{\prime }-l^{\prime }}{l_{B}^{\prime }}p\\ \omega_{1}=\frac{\omega_{1}^{\prime }g}{D} \end{array}\right.$$

The single lens used in the acquisition stage and reconstruction stage of this article is of the same specifications, so *g′* = *g*, *l′* = *D*, and the focal length is *f*, satisfying Gauss’ formula: $\frac{1}{g^{\prime }}+\frac{1}{l^{\prime }}=\frac{1}{g}+\frac{1}{D}=\frac{1}{f}$.

Under optimal lighting conditions, the angular resolution of the human eye (*ε*) ranges from 1′ to 2′ arcminutes. Due to the distribution of photoreceptor cells and their inherent limitations, the human eye’s resolution for 5000 nanometer yellow–green light ranges from 1′ to 2′ arcminutes. Objects wider than 1′ to 2′ arcminutes will blend into the background. The maximum speckle diameter that the human eye can perceive is determined by multiplying the viewing distance by the angular resolution of the eye:

(5) $$\omega_{1}^{\prime }=L\varepsilon$$

The acquisition depth limit of point B on the far object surface can be determined as follows:

(6) $$\Delta l_{B}^{\prime }=\frac{L\varepsilon l^{\prime }}{p-L\varepsilon }$$

Similarly, we can determine the acquisition depth limit of point C on the far side of the object surface in Figure 5.

(7) $$\Delta l_{C}^{\prime }=\frac{L\varepsilon l^{\prime }}{p+L\varepsilon }$$

Therefore, the acquisition depth range of this system can be obtained:

(8) $$\Delta l^{\prime }=\Delta l_{B}^{\prime }+\Delta l_{C}^{\prime }=\frac{2pL\varepsilon l^{\prime }}{p^{2}-L^{2}\varepsilon^{2}}$$

**Figure 6:**
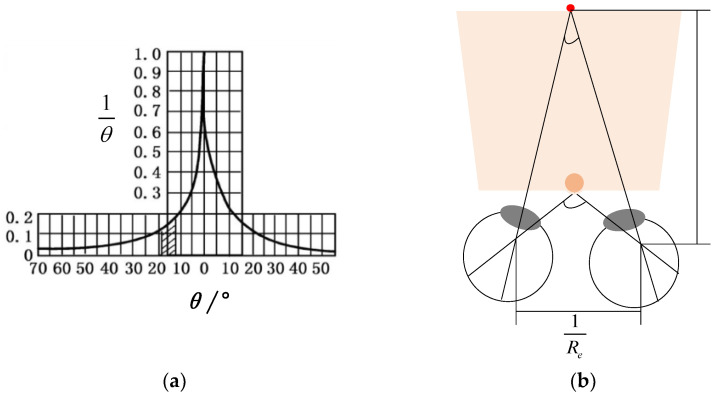
Analysis of visual limits of the human eye.: (**a**) spatial resolution of the human eye; (**b**) line resolution of the human eye.

The minimum angular distance that the human eye can distinguish between two luminous points in space is referred to as the limit resolution angle *θ*. The reciprocal of this is referred to as the angular resolution of the human eye ($\frac{1}{\theta }$). The human eye has an angular resolution range of ±5°. When the range is exceeded, the angular resolution will significantly decrease. In areas beyond ±20° on the retina, the human eye loses its ability to focus and can only perceive the intensity of light [24]. This leads to a blurry retina, as depicted in Figure 6a. In this article, we use *θ* = ± 5°. The line resolution of the human eye in Figure 6b can be expressed as the line resolution:

(9) $$R_{e}=\frac{1}{L\theta }$$

When an image occupies the full range of angles visible to the human eye, it will produce the most optimal visual effect. The optimal viewing distance for the human eye with a fixed display screen of the image is $L_{best}$. For a lens array of m×n dimension, which the diameter of a single lens is *p*, the diagonal length of the displayed image on the display screen is ϕ:

(10) $$L_{best}=\frac{\phi }{2tan\theta }$$

(11) $$\phi =p\sqrt{m^{2}+n^{2}}$$

It can be inferred that the optimal viewing distance is determined when the acquisition and reconstruction of the II system is fixed. The range of depth for acquisition $\Delta l^{\prime }$ is determined using both the distance *l′* of the object being acquired and the angular resolution of the human eye *ε*. In this paper, the value of *ε* is taken as 0.000291 rad.

Let min Z and max Z be the minimum and maximum depths, respectively. During the reconstruction phase, the depth of all reproduced images must fall within this range. The true depth *Z*_(*i*,*j*)_ of pixel (*i*,*j*) must be inversely proportional to the capture distance *l′*_(*i*,*j*)_, which is expressed as:

(12) $$l_{\left(i,j\right)}^{\prime }=\frac{D(maxZ+minZ)}{2Z_{\left(i,j\right)}}$$

According to the Gaussian formula, the true depth *Z*_(*i*,*j*)_ can be obtained as follows:

(13) $$Z_{\left(i,j\right)}=\frac{gf(maxZ+minZ)}{2l_{\left(i,j\right)}^{\prime }(g-f)}$$

According to Equation (8), the range of collected distance *l′*_(*i*,*j*)_, can be obtained.

(14) $$l_{\left(i^{\prime }j\right)}^{\prime }\in \left\{\frac{pl^{\prime }}{\left(p+L\varepsilon \right)},\frac{pl^{\prime }}{\left(p-L\varepsilon \right)}\right\}$$

In order to display all pixels clearly, the reconstructed pixels must be located between the minimum depth at the edge (*min Z* = *D−ΔZ1*) and the maximum depth (*max Z* = *D* + *ΔZ2*). By substituting Equations (2) and (3) into the reproduction formula for the DOF, we obtain the real depth range related to capture distance *l′*:

(15) $$Z_{\left(i,j\right)}\in \left\{\frac{gfD}{g-f}\frac{\left(p-L\varepsilon \right)}{pl^{\prime }}\frac{M^{2}+P_{D}^{2}Dg}{M^{2}-P_{D}^{2}D^{2}},\frac{gfD}{g-f}\frac{\left(p+L\varepsilon \right)}{pl^{\prime }}\frac{M^{2}+P_{D}^{2}Dg}{M^{2}-P_{D}^{2}D^{2}}\right\}$$

The system parameter $M=gp(1+\frac{g^{\prime }}{l^{\prime }})$ relies on the inherent parameters of the display system, which can be determined using the Gaussian formula. Finally, starting with the visual characteristics of the human eye, we analyze the path of light from a point source to the eye in the acquisition and reconstruction of light fields. Based on the reproduction DOF model obtained in the previous section, we derive a target acquisition DOF model that is related to the acquisition distance. We are ultimately developing an overall DOF model of II based on the HVS.

## 3. Research of II System Based on the Human Visual Distance (HVD) Perception Model

### 3.1. Research on Generating EIA Based on the HVD Perception

We obtained a depth range model affected by the collection distance through deduction. Some targets outside the depth range may experience phenomena such as image blur and low resolution during reconstruction. Next, we will further design the EIA generation method. This method can ensure a large DOF while improving the viewing resolution after reconstruction. Now let us introduce this EIA design method.

**Figure 7:**
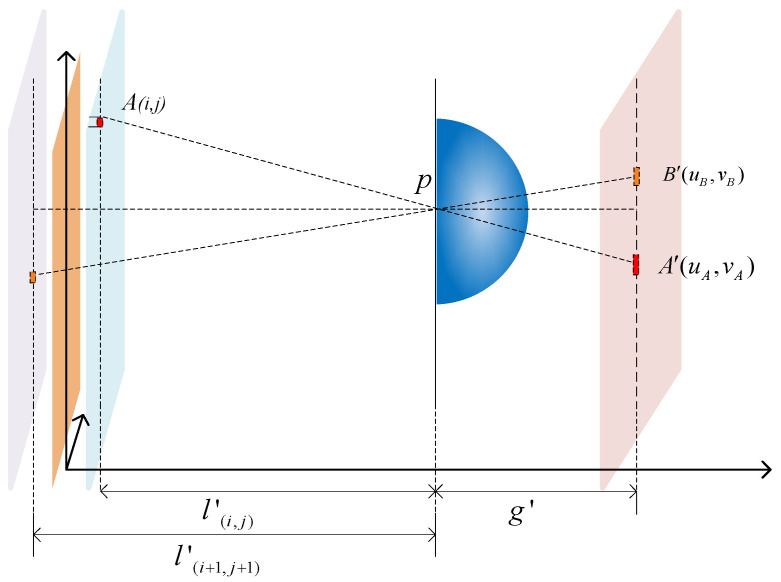
Analysis of pixel acquisition.

Figure 7 illustrates the geometric relationship between the object pixels projected onto the EIA through a unit lens. The light rays from object pixels *A*_(*i*,*j*)_ and *B*_(*i*,*j*)_ pass through the lens center and form image points *A′*_(*i*,*j*)_ and *B′*_(*i*,*j*)_ on EIA. The pixel co-ordinates (*u*, *v*) are as follows:

(16) $$\left\{\begin{array}{l} u=p\cdot i_{m}-(i\cdot P_{I}-p\cdot i_{m})\cdot \frac{g^{\prime }}{l_{\left(i,j\right)}^{\prime }}\\ v=p\cdot j_{m}-(j\cdot P_{I}-p\cdot j_{m})\cdot \frac{g^{\prime }}{l_{\left(i,j\right)}^{\prime }} \end{array}\right.$$

where *i* and *j* represent the pixel indices on the *x* and *y* axes of an object, and *i_m_* and *j_m_* represent the indices of the lens on the *x* and *y* axes. By using Equation (16), we can calculate the position *A′*_(*uA*, *vA*)_ on the EIA that corresponds to any point *A*_(*i*,*j*)_ in the light field. This allows us to obtain the EIA corresponding to lens m.

**Figure 8:**
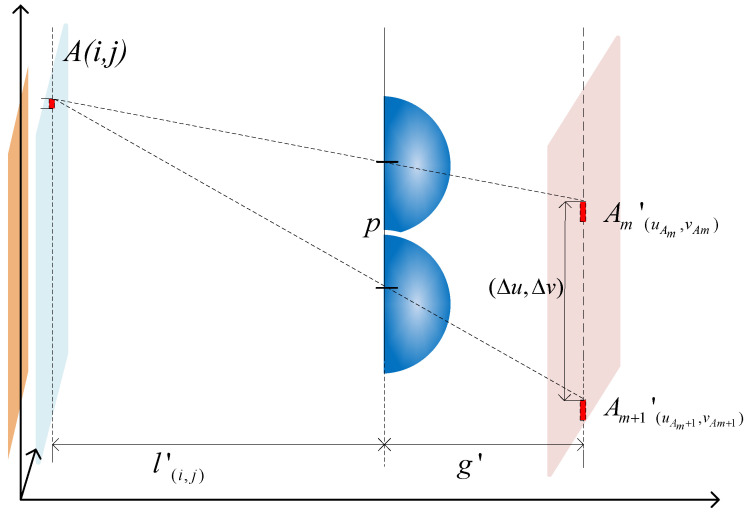
Displacement relationship of homonymous image points during the collection process.

Figure 8 shows the displacement relationship of the homonymous image points obtained on the EIA after point A passes through lens m and lens *m* + 1:

(17) $$\Delta u=\Delta v=p^{2}(1+\frac{g^{\prime }}{l_{\left(i,j\right)}^{\prime }})$$

**Figure 9:**
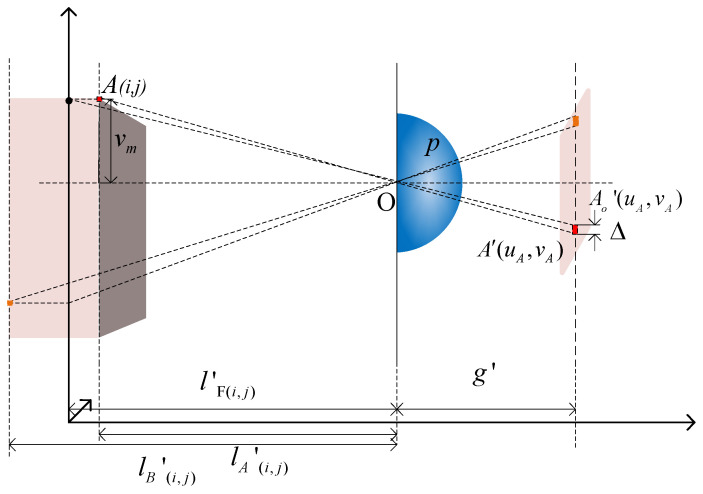
Analysis of pixel calibration.

The co-ordinates of the homonymous image points obtained from different lenses can be determined using the formula mentioned above. However, the pixels in EIA need additional calibration. Figure 9 shows the correction analysis of the object point A.

The object point A is imaged as *A′* on the unit element image through micro-lens *m*. The horizontal corresponding point of object point A on the focused object surface is *A_O_*, and its corresponding image point on the unit element image is *A_O_′*. The vertical distance between *A′* and *A_O_′* is Δ, meeting the condition: $\Delta \geq P_{D}$. It is necessary to adjust the mapping co-ordinates of the object pixels. *P_D_* represents the pixel size of the display device, and Δ can be calculated using geometric relationships:

(18) $$\Delta =v_{0}g^{\prime }(\frac{1}{l_{A(i,j)}^{\prime }}-\frac{1}{l_{F(i,j)}^{\prime }})$$

Since the object point may be located at either a far object point or a near object point position, the final correction condition of the pixel acquisition model can be obtained based on the real depth data *l′*_(*i*,*j*)_ of the object point:

(19) $$\left\{\begin{array}{l} \left|\frac{1}{l_{\left(i,j\right)}^{\prime }}-\frac{1}{l_{F(i,j)}^{\prime }}\right|\geq \frac{P_{D}}{g^{\prime }u_{m}}\\ \left|\frac{1}{l_{\left(i,j\right)}^{\prime }}-\frac{1}{l_{F(i,j)}^{\prime }}\right|\geq \frac{P_{D}}{g^{\prime }v_{m}} \end{array}\right.$$

The distance from point A to the center of the lens, denoted as $l_{F(i,j)}^{\prime }$, can be calculated using the Gaussian formula. The variables *u_m_* and *v_m_* represent the maximum distance offsets of point A in the horizontal and vertical directions, respectively, from the center *O* of the micro-lens array *m* and the corresponding lens surface.

By substituting the range of collected distance from the previous section (Equation (14)) into Equation (19) of the pixel capture model correction, we finally derive the perceived visual range limited by human eye visual characteristics:

(20) $$L\geq max\left(\frac{P_{D}l_{F(i,j)}^{\prime }p}{g^{\prime }\varepsilon u_{m}},\frac{P_{D}l_{F(i,j)}^{\prime }p}{g^{\prime }\varepsilon v_{m}}\right)$$

By substituting into Equation (9), we can determine the threshold of the line resolution of the human eye.

(21) $$R_{e-SCL}=min\left(\frac{g^{\prime }\varepsilon u_{m}}{P_{D}l_{F(i,j)}^{\prime }p\theta },\frac{g^{\prime }\varepsilon v_{m}}{P_{D}l_{F(i,j)}^{\prime }p\theta }\right)$$

where *ε* is the angular resolution of the human eye, and *θ* is the minimum resolution angle of the human eye.

The above deduction suggests that the display effect of the reconstruction stage of the II system is limited by two conditions. The first condition is that the 3D objects must be within the real capture distance range *l′*_(*i*,*j*)_, as specified by Equation (14). Therefore, based on the capture range model, we can achieve a clear image of the entire scene by adjusting the DOF for 3D objects which is outside the range of real capture depth.

The second condition is that the viewing distance, *L*, must be within the range of visual perception. For a 3D object within the real acquisition distance range *l′*_(*i*,*j*)_, as long as the viewing distance *L* is within the range of visual perception, the visual characteristics of the human eye have little impact on imaging. Therefore, each pixel in the unit element image does not require correction and can directly capture EIA images. When the viewing distance *L* reaches the critical position for visual perception, the line resolution of the human eye reaches its threshold, resulting in the clearest image perceived by the human eye. However, if the viewing distance (*L*) is smaller than the critical position of visual perception, the human eye’s visual characteristics will have a greater impact on imaging. The current *l′*_(*i*,*j*)_ no longer meets the target acquisition DOF model derived in Section 2.2. However, the pixel acquisition model must still satisfy the correction condition in Equation (19). Therefore, when the human eye’s viewing distance (*L*) is smaller than the critical position of visual perception, the adjustment range for the acquisition distance become *l″*_(*i*,*j*)_:

(22) $${l^{\prime \prime }}_{\left(i,j\right)}\in \left\{\begin{array}{l} max\left(\frac{g^{\prime }u_{m}}{g^{\prime }u_{m}+P_{D}l_{F(i,j)}^{\prime }},\right.\left.\frac{g^{\prime }v_{m}}{g^{\prime }v_{m}+P_{D}l_{F(i,j)}^{\prime }}\right),\\ min\left(\frac{g^{\prime }u_{m}}{g^{\prime }u_{m}-P_{D}l_{F(i,j)}^{\prime }},\frac{g^{\prime }v_{m}}{g^{\prime }v_{m}-P_{D}l_{F(i,j)}^{\prime }}\right) \end{array}\right\}$$

Therefore, if the human eye’s viewing distance (*L*) is smaller than the critical position of visual perception, correcting the EIA based on new real depth data can greatly enhance image quality.

### 3.2. Design of Interactive II Display System

After acquiring the human eye visual perception model, we developed an interactive II display system. We developed a face–eye tracking model for traditional II display systems. This model utilizes the YOLOv5 network, imported through the DNN module for face and eye detection [25]. The system can detect faces and identify the positions of eye landmarks. The YOLOv5 network consists of backbone, neck, and head, and it uses a feature pyramid network (FPN) to merge the feature layers of different shapes, enhancing feature extraction and leading to accurate and robust detection results.

By capturing faces through the camera, we accurately located the positions of faces and eyes, and then used a binocular camera setup for precise real-time distance measurement. Finally, we employed TensorRT for model inference, achieving a detection frame rate of at least 24 frames per second, which meets real-time requirements.

**Figure 10:**
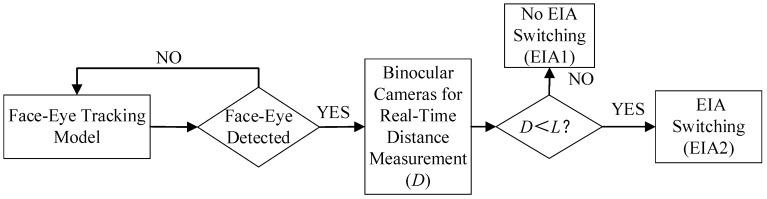
Workflow of the interactive II display system.

The workflow of the interactive system is illustrated in Figure 10. When the human eye detection model fails to detect any face or eyes within the imaging observation range, the display screen will directly show the uncalibrated EIA1. EIA1 is obtained by satisfying the acquisition distance in Equation (14). When the human eye detection model identifies eyes within the imaging observation range, it activates the real-time retrieval of depth information from the binocular camera, allowing for real-time distance measurement (*D*). The information is then sent back to the visual perception model, enabling alteration of the display within different ranges of visual perception. When *D* is greater than the actual viewing distance (*L*), the display screen still shows EIA1 and there is no EIA switching. When *D* is less than *L*, the display will perform EIA switching and display EIA2. This EIA2 is obtained after calibration of the acquisition distance according to Equation (22).

## 4. Experimental Results

**Figure 11:**
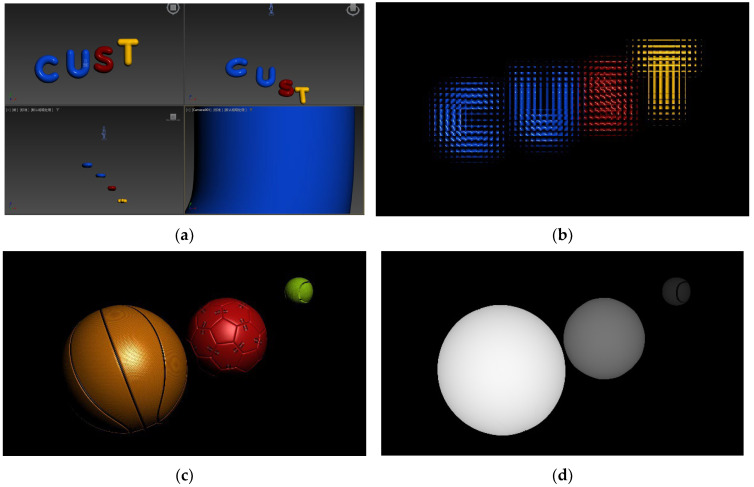
Building image collection scene with 3Ds Max: (**a**) 3Ds Max simulated pixel collection scene; (**b**) collected EIA; (**c**) collected RGB image; (**d**) collected depth image.

**Figure 12:**
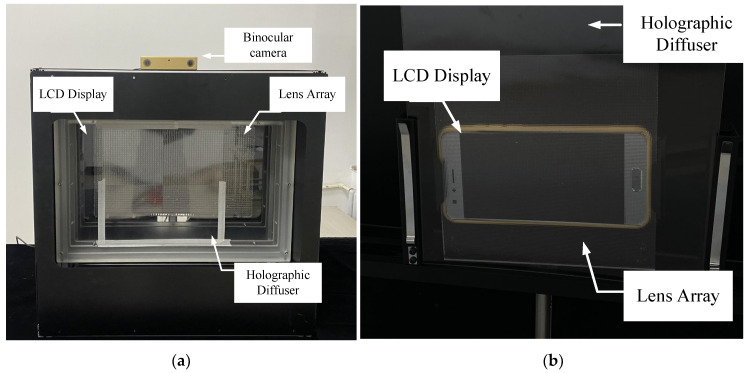
Optical reconstruction experimental platform: (**a**) optical experimental platform 1; (**b**) optical experimental platform 2.

In order to validate the proposed model, 3Ds Max, a 3D modeling software, was used to create the models for the pixel collection scenes (Figure 11a). The collected EIA is depicted in Figure 11b–d. Two sets of experiments were conducted to validate the proposed overall DOF model of II based on HVS and the HVD perception model in this study. The experimental platform used for optical reconstruction in this paper is depicted in Figure 12a,b and the corresponding parameters are listed in Table 1.

### 4.1. Experimental Validation of the II Overall DOF Model Based on HVS

The collection process involved the direct use of 3Ds Max to collect EIA with a micro-lens array, as shown in Figure 11a. The collected EIA is shown in Figure 11b. The optical experiments were conducted using Optical Experimental Platform 1, as shown in Figure 12a, with the relevant parameters provided in Table 1. Based on the parameters and model formulas from the table, the relevant parameters for the overall DOF of II based on the HVS can be obtained, as shown in Table 2.

**Figure 13:**
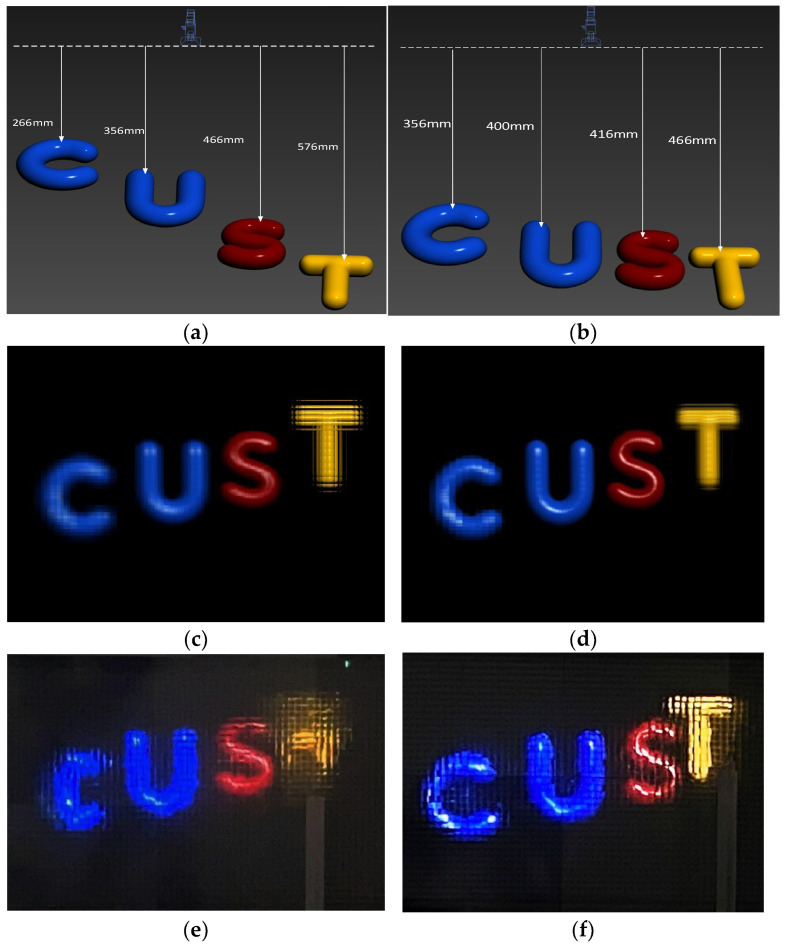
Overall DOF model verification experiment: (**a**) Collection Scene 1; (**b**) Collection Scene 2; (**c**) computer reconstruction of Scene 1; (**d**) computer reconstruction of Scene 2; (**e**) optical experiment of Scene 1; (**f**) optical experiment of Scene 2.

Based on the relevant parameters of the overall DOF model, the lens array and the 3D model were arranged as shown in Figure 13a,b, representing Scene 1 and Scene 2. Two sets of EIA were collected: in Figure 13a, the letter “C” and “T” are outside the captured depth range, while in Figure 13b, the letter “C” and “T” are within the captured depth range. Figure 13c,d shows the computer reconstructions at the central depth plane *D* = 403.96 mm for the two capture scenes [26], while Figure 13e,f display the optical experimental results.

The optical experimental results align with the simulation results. All reconstructed letters within the capture depth range have distinct outlines and smooth model edges. The reconstruction image shows blurred contours and significant distortion for the letters “C” and “T” located outside the capture depth range. Therefore, within the capture depth range, the reconstruction results of the obtained EIA are clear and in focus under this capture model.

**Figure 14:**
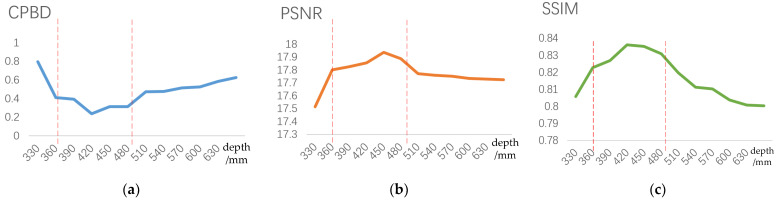
Objective evaluations at different positions of reconstruction distance: (**a**) CPBD for reconstructed images; (**b**) SSIM for reconstructed images; (**c**) PSNR for reconstructed images.

Various objective evaluations were conducted on computer reconstruction images at different distances. Figure 14a depicts the cumulative probability of blur detection (CPBD) using blue-light detection [27], an objective evaluation method that does not rely on a reference image. The lower the CPBD, the less blurry the current image’s graphic edges. The structural similarity index (SSIM) and peak signal-to-noise ratio (PSNR) are commonly used indicators for comparing the similarity between two images. These two metrics both require a reference image. Figure 14b illustrates the SSIM between the two images. Its value range is between 0 and 1. The SSIM measures the similarity between two images. A value closer to 1 indicates a higher similarity in the structure of the images. Figure 14c displays the PSNR of the two images. The higher the PSNR value, the lower the distortion and similarity. A PSNR value above 40 dB indicates that the image quality is very similar to the reference image. A value of 30 and 40 dB typically indicates better image quality. The distortion is perceptible but acceptable when the PSNR is between 30 and 40 dB. Poor image quality is indicated when the PSNR is between 20 and 30 dB, and a PSNR lower than 20 dB is considered unacceptable. All three evaluation methods are in line with the characteristics of the human visual system. The graphs show that for the reconstructed three-dimensional images at a depth of 360–480 mm, the CPBD value is below 41%, the SSIM value is above 82%, and the PSNR value is above 17.80. This suggests that the reconstructed images have distinct outlines, closely resemble the original model in structure, and demonstrate improved quality in reconstruction. This conclusion aligns with the *Z*_(*i*,*j*)_ data presented in Table 2 and the earlier deductions made in the text.

**Figure 15:**
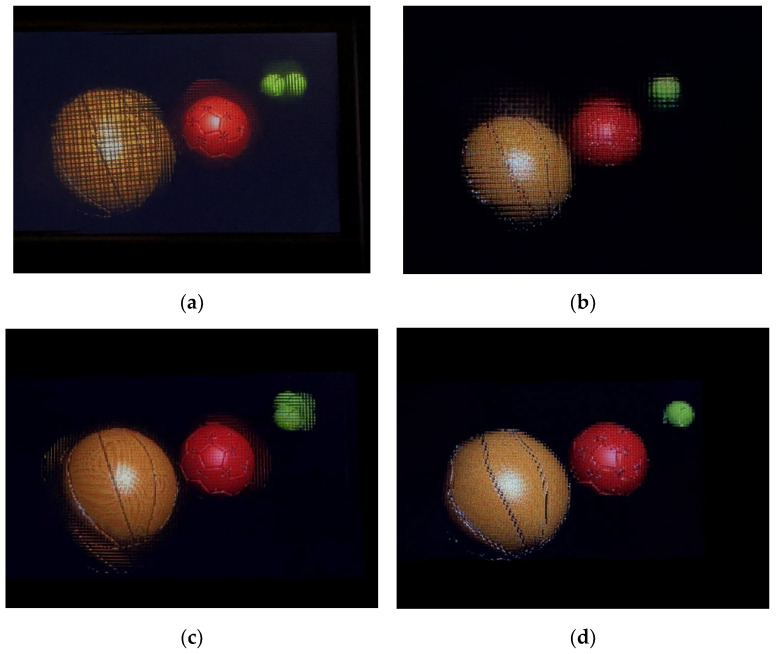
Optical reconstruction experimental results: (**a**) RODC algorithm [28]; (**b**) RIOP algorithm [29]; (**c**) LFR algorithm [30]; (**d**) our method.

Next, we compared our method with three EIA generation algorithms that do not take into account the capture depth [28,29,30]. A virtual depth camera was used to capture color images and the corresponding depth images, which served as the raw data for generating the EIA (Figure 11c,d). Optical experiments were conducted using the apparatus depicted in Figure 12b, with the relevant parameters provided in Table 1—Optical Experimental Platform 2. The optical experimental results are shown in Figure 15. Both the RODC algorithm and RIOP algorithm have poor reconstruction effects for yellow and green balls outside the depth of field. These two methods are not effective in reconstructing 3D scenes that are beyond the DOF. The LFR algorithm is effective in reconstructing objects with smaller depths (yellow and red spheres) clearly, but it struggles to maintain clarity for objects with larger depths (green sphere). Our algorithm successfully achieves clear image reconstruction across the entire depth field. The image edges are sharper, and the spatial resolution of the images is significantly improved.

Then, we compare the objective evaluations of our method with the other three methods, shown in Table 3. It is clear that our approach produces better results both in optical experiments and objective evaluations: the CPBD value of our method is 38.73%, the SSIM value of our method is 86.56%, and the PSNR value of our method is 31.12. Our method achieved the best results in these three objective evaluation comparisons, and it is aligned with the subjective evaluation shown in Figure 15.

### 4.2. Verification Experiment of the HVD Perception Model

After validating the reasonableness of the overall DOF model of II base on HVS proposed in this paper, we conducted further experiments to validate the reasonableness of the HVD perception mode. The reconstruction experiments were conducted using Optical Experimental Platform 1. Based on the spatial position of the 3D model, the relevant parameters, visual perception range, and corresponding capture depth were determined and are shown in Table 4.

**Figure 16:**
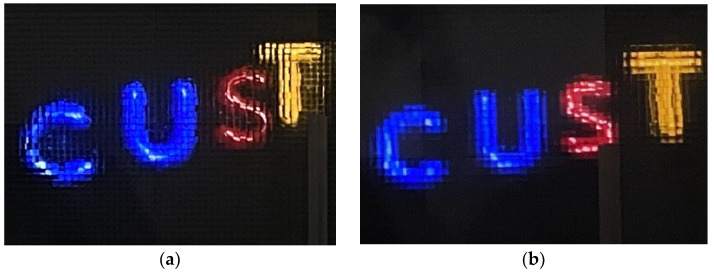
Optical reconstruction results of two types of pixels at *L* = 2 m.: (**a**) optical experiment before improvement; (**b**) optical experiment after improvement.

After improvement based on the HVD perception model, different EIAs are generated. The optical reconstruction results of different pixels before and after improvement when the viewing distance (*L*) is less than the critical distance of visual perception (*L* = 2 m) are shown in Figure 16. The initial image quality of the EIA (Figure 16a) is poor, with noticeable light ray crosstalk issues, blurry edges, and low resolution. This does not align with human visual perception. The image quality of the improved EIA (Figure 16b) has significantly improved. There are no visible issues with light ray crosstalk, and the image edges are clear. The quality of the optical reconstruction has improved to match human visual characteristics.

**Figure 17:**
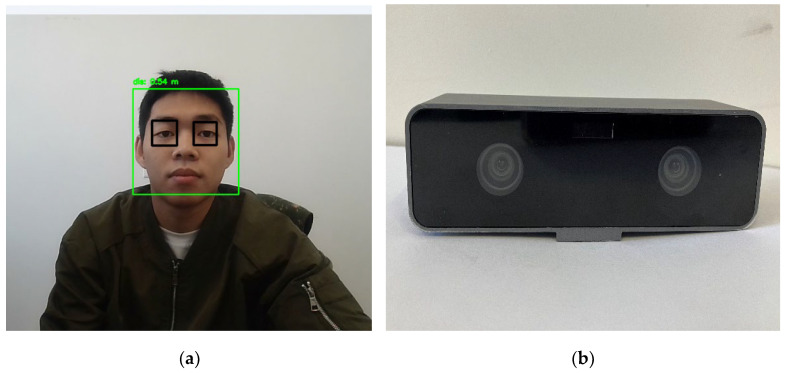
Human face–eye detection and distance measurement device: (**a**) visual distance detection results; (**b**) custom binocular camera.

On the basis of the HVD perception mode, we used the interactive II display system as described in Section 3.2. The face–eye detection model used can output the face detection box and the positions of eye landmarks (see Figure 17a). We achieved accurate real-time distance measurements using a custom binocular camera (Figure 17b). The resolution is 1280 × 720, with a maximum image transfer rate of 30 frames per second (FPS).

**Figure 18:**
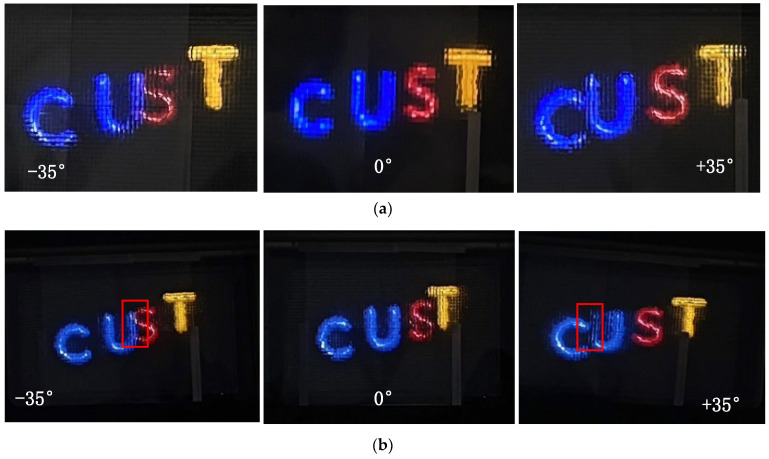

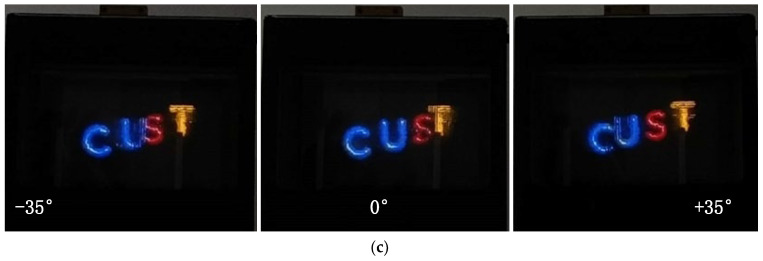
Optical reconstruction at various viewing distances after improvement using the HVD perception model: (**a**) *L* = 2 m; (**b**) *L* = 2.74 m; (**c**) *L* = 4 m.

We conducted optical experiments using our interactive II display system. Figure 18 shows the optical reconstruction results at various viewing distances. It is evident that the image remains clear and the resolution improves as the viewing distance changes from far to near. Each integral imaging system has a limit to its field of view. If the viewing angle exceeds this limit to its field of view, the reconstructed image may become distorted. The field of view limit in Figure 18 of this article is ±35°. Therefore, images at the extreme field of view may produce noise, as shown in the left and right images in Figure 18a. However, the noise in the images at ±35° are still considered acceptable. The details shown in Figure 18b indicate that there is noticeable parallax in the letter models when viewed from different positions, suggesting a greater depth of field. Our method leads to a stable transition of three-dimensional images from various viewpoints, resulting in an overall enhancement in the quality of light field reconstruction.

With the interactive II display system that we designed, users can switch the EIA between different ranges of visual perception, enhancing the overall viewing experience of the II (Figure 18). Even within a visual perception range of less than 2.74 m, the human eye can still view content clearly. This method enhances human eye perception by extending its range, maintaining spatial resolution, improving depth of field in reconstruction, and significantly enhancing the display effectiveness of II.

## 5. Conclusions

This research discusses two issues in the II system: the unclear reconstruction of targets beyond the acquisition range, and the unclear imaging of 3D object within a certain range when observed with the human eye. First, the overall DOF model of an II system based on the HVS that we proposed has a guiding role in solving the reconstruction problem of fuzzy targets that are outside the DOF range. On this basis, we conducted further analysis and developed a method of generating EIA based on HVD perception. This method solves the problem of unclear images when observing outside the visual distance. Finally, the interactive II display system designed in this article achieves a real-time switching effect of EIA in various observation ranges. This work not only enhances the observable range of the human eye in integral imaging, but also has a positive impact on improving the DOF and viewing resolution. Ultimately, it enhances the overall effect of light field reconstruction. Since the main focus of this study is to enhance the DOF and viewing resolution, future research will aim to improve the field of view, thereby significantly enhancing the overall viewing effect of integral imaging and creating a 3D display effect that closely resembles a real scene.

## Figures and Tables

**Table 1:** Parameters of the II system.

Parameters	Optical Experimental Platform 1	Optical Experimental Platform 2
Lens’ focal length (*f*)	36.76 mm	3.00 mm
Lens’ diameter (*p*)	8.50 mm	1.00 mm
Lens array (*m* × *n*)	70 × 39	112 × 63
Distance between lens and display (*g*)	40.44 mm	3.60 mm
Distance between lens and CDP (*D*)	403.96 mm	18.00 mm
EIA resolution	3818 × 2127	1904 × 1071
Resolution of display	3840 × 2160	1920 × 1080
Pixel size of display (*P_D_*)	0.1558 mm	0.0588 mm
Optimal viewing distance (*L_best_*)	3.89 m	0.92 m

**Table 2:** Parameters for the overall DOF of II based on the HVS.

Parameters	Optical Experimental Platform 1	Optical Experimental Platform 2
Focal Plane (*l′*)	403.96 mm	18.00 mm
Captured Depth Range (Δ*l′*)	109.54 mm	11.30 mm
Captured Distance Range (*l′*_(*i*,*j*)_)	356.48–466.02 mm	13.70–25.00 mm
Real Depth Range Related to Captured Distance (*Z*_(*i*,*j*)_)	361.01–471.95 mm	14.20–25.47 mm

**Table 3:** Objective evaluations of different methods.

Methods	CPBD	PSNR	SSIM
RODC algorithm [28]	0.7904	19.79	0.2313
RIOP algorithm [29]	0.6700	20.32	0.6186
LFR algorithm [30]	0.4619	29.93	0.7726
Our method	0.3873	31.12	0.8656

**Table 4:** Parameters for the HVD perception model.

Parameters	Optical Experimental Platform 1
Horizontal distance offset of the 3D object from the center O of the micro-lens array (*u*)	(−25 mm, +25 m)
Vertical distance offset of the 3D object from the center O of the micro-lens array (*v*)	(−16.6 mm, +16.66 m)
Visual perception range (*L*)	*L* ≥ 2.74 m
The threshold of visual line resolution of the human eye ($R_{e-SCL}$)	4.18 × 10^−3^ (mm^−1^)
Collection distance range when *L* ≥ 2.74 m (*l_1′_*_(*i*,*j*)_)	356.48–466.02 mm
Adjustment range of collection distance when *L* < 2.74 m (*l″*_(*i*,*j*)_)	−0.91–−1.10 mm
Collection distance range when *L* < 2.74 m (*l_2′_*_(*i*,*j*)_)	355.57–464.92 mm

## Data Availability

Not applicable.
